# Supplementary material for: The effects of the sex chromosomes on the inheritance of species-specific traits of the copulatory organ shape in Drosophila virilis and Drosophila lummei
Source: PLoS One. 2020 Dec 29;15(12):e0244339. doi: 10.1371/journal.pone.0244339 (PMC7771703; doi:10.1371/journal.pone.0244339)
Supplement: S1 Fig — The genotypes are abbreviated as in Table 2. The chromosomes and paternal genotype are indicated in the following order: X chromosome, Y chromosome, autosomes, male parent identity. (DOCX) [file pone.0244339.s001.docx]

S1 Fig 1. Genotype distribution in the space of the first two principal components.


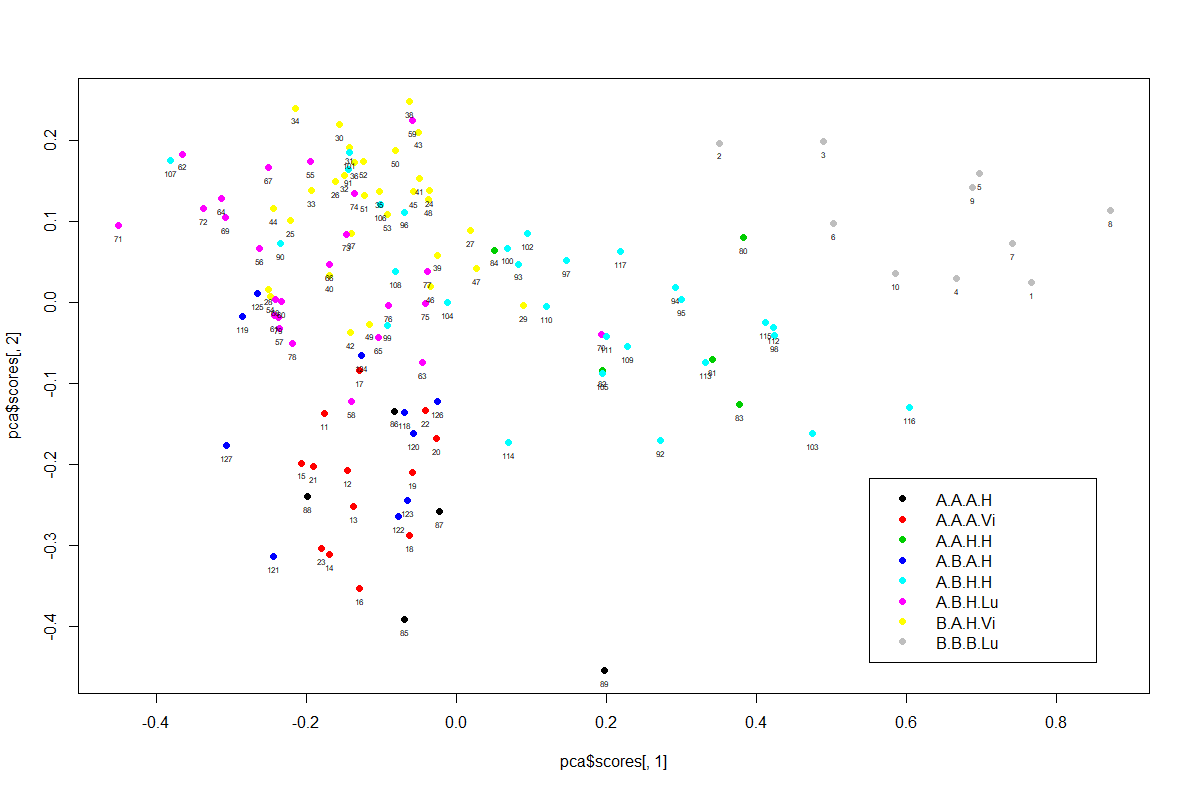


The genotypes are abbreviated as in Table 2. The chromosomes and paternal genotype are indicated in the following order: X chromosome, Y chromosome, autosomes, male parent identity.
