# Supplementary material for: The effects of the sex chromosomes on the inheritance of species-specific traits of the copulatory organ shape in Drosophila virilis and Drosophila lummei
Source: PLoS One. 2020 Dec 29;15(12):e0244339. doi: 10.1371/journal.pone.0244339 (PMC7771703; doi:10.1371/journal.pone.0244339)
Supplement: S1 Table — The sample size is indicated in parentheses. M, mean; σ, standard deviation. Superscripts in the first column indicate that the trait is incorporated with a high weight in the respective factor structure. (DOCX) [file pone.0244339.s002.docx]

S1 Table. Variation of the morphometric traits in the shape of the male copulatory system.

| Signs | 0.0.4 (10) | | 1.0.8 (31) | | 1.0.9 (25) | | 2,8,1 (5) | | 2,8,13 (5) | | 2,9,1 (28) | | 2,9,30 (10) | | vir160 (13) | |
| --- | --- | --- | --- | --- | --- | --- | --- | --- | --- | --- | --- | --- | --- | --- | --- | --- |
|  | M | σ | M | σ | M | σ | M | σ | M | σ | M | σ | M | σ | M | σ |
| IMP2^3,6^ | 1.023 | 0.013 | 0.998 | 0.018 | 1.008 | 0.010 | 1.021 | 0.013 | 1.058 | 0.047 | 1.019 | 0.020 | 1.028 | 0.012 | 1.019 | 0.010 |
| IMP3 | 0.110 | 0.012 | 0.098 | 0.017 | 0.106 | 0.017 | 0.123 | 0.017 | 0.066 | 0.010 | 0.100 | 0.017 | 0.086 | 0.022 | 0.076 | 0.020 |
| IMP4^3^ | 0.151 | 0.017 | 0.153 | 0.019 | 0.150 | 0.018 | 0.145 | 0.025 | 0.188 | 0.025 | 0.159 | 0.023 | 0.191 | 0.016 | 0.175 | 0.021 |
| IMP5^7^ | 0.023 | 0.012 | 0.018 | 0.013 | 0.020 | 0.013 | 0.025 | 0.014 | 0.017 | 0.006 | 0.009 | 0.007 | 0.024 | 0.008 | 0.025 | 0.007 |
| IMP6^3^ | 0.180 | 0.020 | 0.201 | 0.030 | 0.203 | 0.021 | 0.190 | 0.022 | 0.328 | 0.061 | 0.218 | 0.041 | 0.298 | 0.042 | 0.259 | 0.041 |
| IMP7^7^ | 0.066 | 0.011 | 0.040 | 0.024 | 0.050 | 0.008 | 0.050 | 0.020 | 0.034 | 0.026 | 0.038 | 0.013 | 0.064 | 0.040 | 0.052 | 0.024 |
| IMP8^5^ | 0.287 | 0.019 | 0.372 | 0.044 | 0.381 | 0.034 | 0.329 | 0.028 | 0.381 | 0.034 | 0.387 | 0.046 | 0.381 | 0.037 | 0.368 | 0.023 |
| IMP9^7^ | 0.057 | 0.019 | 0.030 | 0.033 | 0.047 | 0.018 | 0.013 | 0.009 | 0.008 | 0.020 | 0.018 | 0.026 | 0.022 | 0.029 | 0.011 | 0.029 |
| IMP10^5^ | 0.352 | 0.044 | 0.350 | 0.062 | 0.346 | 0.050 | 0.286 | 0.046 | 0.215 | 0.034 | 0.298 | 0.046 | 0.234 | 0.047 | 0.230 | 0.026 |
| IMP11^4^ | 0.087 | 0.021 | 0.113 | 0.036 | 0.054 | 0.029 | 0.088 | 0.015 | 0.042 | 0.023 | 0.061 | 0.031 | 0.058 | 0.046 | 0.061 | 0.020 |
| IMP12^4^ | 0.145 | 0.031 | 0.124 | 0.029 | 0.128 | 0.026 | 0.106 | 0.014 | 0.119 | 0.019 | 0.120 | 0.022 | 0.173 | 0.171 | 0.115 | 0.030 |
| IMP13^5^ | 0.397 | 0.037 | 0.431 | 0.032 | 0.428 | 0.032 | 0.366 | 0.020 | 0.402 | 0.034 | 0.400 | 0.039 | 0.413 | 0.027 | 0.417 | 0.043 |
| IMP14^3^ | 0.273 | 0.051 | 0.356 | 0.057 | 0.359 | 0.048 | 0.306 | 0.058 | 0.505 | 0.016 | 0.353 | 0.056 | 0.477 | 0.056 | 0.472 | 0.046 |
| IMP15^5^ | 0.372 | 0.035 | 0.405 | 0.030 | 0.405 | 0.030 | 0.360 | 0.014 | 0.383 | 0.034 | 0.402 | 0.035 | 0.419 | 0.047 | 0.380 | 0.028 |
| IMP16^3^ | 0.362 | 0.037 | 0.447 | 0.070 | 0.467 | 0.051 | 0.475 | 0.077 | 0.689 | 0.054 | 0.503 | 0.068 | 0.633 | 0.035 | 0.591 | 0.033 |
| IMP17^7^ | 0.084 | 0.018 | 0.045 | 0.014 | 0.063 | 0.011 | 0.058 | 0.015 | 0.036 | 0.023 | 0.046 | 0.020 | 0.054 | 0.014 | 0.054 | 0.024 |
| IMP18 | 0.624 | 0.070 | 0.657 | 0.084 | 0.619 | 0.053 | 0.745 | 0.050 | 0.747 | 0.160 | 0.669 | 0.057 | 0.717 | 0.071 | 0.732 | 0.071 |
| IMP19 | 0.176 | 0.020 | 0.229 | 0.041 | 0.194 | 0.028 | 0.236 | 0.026 | 0.289 | 0.040 | 0.204 | 0.042 | 0.241 | 0.061 | 0.245 | 0.054 |
| IMP20^4^ | 0.107 | 0.019 | 0.105 | 0.026 | 0.062 | 0.018 | 0.092 | 0.016 | 0.045 | 0.023 | 0.073 | 0.028 | 0.060 | 0.030 | 0.067 | 0.021 |
| IMP21^3^ | 0.073 | 0.006 | 0.077 | 0.012 | 0.080 | 0.015 | 0.076 | 0.018 | 0.082 | 0.004 | 0.076 | 0.011 | 0.098 | 0.010 | 0.085 | 0.009 |
| IMP22 | 0.025 | 0.011 | 0.032 | 0.020 | 0.029 | 0.014 | 0.027 | 0.010 | 0.023 | 0.010 | 0.024 | 0.025 | 0.017 | 0.011 | 0.026 | 0.015 |
| IMP23 | 0.171 | 0.016 | 0.191 | 0.037 | 0.195 | 0.023 | 0.236 | 0.025 | 0.208 | 0.030 | 0.205 | 0.030 | 0.240 | 0.034 | 0.192 | 0.028 |
| IMP24 | 0.169 | 0.033 | 0.240 | 0.057 | 0.239 | 0.061 | 0.187 | 0.036 | 0.308 | 0.086 | 0.184 | 0.058 | 0.206 | 0.073 | 0.259 | 0.069 |
| IMP25^3^ | 0.163 | 0.029 | 0.247 | 0.052 | 0.294 | 0.033 | 0.218 | 0.036 | 0.392 | 0.022 | 0.251 | 0.049 | 0.356 | 0.051 | 0.344 | 0.052 |
| IMP26 | 0.069 | 0.015 | 0.074 | 0.029 | 0.071 | 0.026 | 0.078 | 0.023 | 0.097 | 0.014 | 0.080 | 0.020 | 0.103 | 0.031 | 0.089 | 0.015 |
| IMP27 | 0.162 | 0.041 | 0.171 | 0.022 | 0.187 | 0.020 | 0.184 | 0.014 | 0.198 | 0.025 | 0.176 | 0.024 | 0.209 | 0.037 | 0.181 | 0.021 |
| IMP28 | 0.606 | 0.075 | 0.581 | 0.103 | 0.655 | 0.064 | 0.638 | 0.021 | 0.571 | 0.065 | 0.593 | 0.074 | 0.578 | 0.108 | 0.695 | 0.115 |
| IMP29^1^ | 0.068 | 0.021 | 0.063 | 0.014 | 0.059 | 0.010 | 0.053 | 0.009 | 0.063 | 0.006 | 0.062 | 0.011 | 0.056 | 0.012 | 0.059 | 0.008 |
| IMP30^2^ | 0.049 | 0.021 | 0.039 | 0.024 | 0.078 | 0.029 | 0.053 | 0.021 | 0.057 | 0.029 | 0.042 | 0.017 | 0.046 | 0.009 | 0.081 | 0.046 |
| IMP31^1^ | 0.048 | 0.006 | 0.052 | 0.014 | 0.050 | 0.008 | 0.043 | 0.002 | 0.045 | 0.005 | 0.046 | 0.007 | 0.047 | 0.004 | 0.049 | 0.007 |
| IMP32^2^ | 0.103 | 0.033 | 0.052 | 0.032 | 0.109 | 0.043 | 0.082 | 0.030 | 0.087 | 0.036 | 0.063 | 0.026 | 0.051 | 0.016 | 0.088 | 0.044 |
| IMP33^1^ | 0.037 | 0.006 | 0.052 | 0.018 | 0.046 | 0.004 | 0.035 | 0.004 | 0.044 | 0.002 | 0.043 | 0.008 | 0.050 | 0.003 | 0.047 | 0.009 |
| IMP34^2^ | 0.103 | 0.038 | 0.034 | 0.030 | 0.080 | 0.035 | 0.070 | 0.033 | 0.073 | 0.024 | 0.048 | 0.028 | 0.030 | 0.022 | 0.068 | 0.031 |
| alpha^6^ | 0.353 | 0.158 | 1.070 | 0.089 | 1.127 | 0.140 | 0.688 | 0.151 | 0.911 | 0.154 | 0.817 | 0.249 | 1.059 | 0.113 | 1.018 | 0.059 |
| beta^2^ | 0.194 | 0.112 | 0.250 | 0.101 | 0.371 | 0.115 | 0.293 | 0.061 | 0.286 | 0.133 | 0.239 | 0.114 | 0.239 | 0.128 | 0.434 | 0.115 |

The sample size is indicated in parentheses. M, mean; σ, standard deviation. Superscripts in the first column indicate that the trait is incorporated with a high weight in the respective factor structure.
