# Supplementary material for: The effects of the sex chromosomes on the inheritance of species-specific traits of the copulatory organ shape in Drosophila virilis and Drosophila lummei
Source: PLoS One. 2020 Dec 29;15(12):e0244339. doi: 10.1371/journal.pone.0244339 (PMC7771703; doi:10.1371/journal.pone.0244339)
Supplement: S2 Table — (DOCX) [file pone.0244339.s003.docx]

S2 Table. Dominance at traits of the system of copulatory shape as dependent on the sex chromosome composition in *D. virilis/D. lummei* hybrid males homozygous for the *D. virilis* autosomes.

| Factor | Sign | ♂F_1_(♂_Lu_ x♀_Vi_) × ♀_Vi_ | | ♂F_1_(♂_Vi_ x♀_Lu_) × ♀_Vi_ | |
| --- | --- | --- | --- | --- | --- |
|  |  | F_b_ X_Vi_Y_Lu_ **2.9.30** | | F_b_ X_Vi_Y_Vi_ **2.8.13** | |
|  |  | D_x_ | P.-h. | D_x_ | P.-h. |
| F1 | imp33 | D_Vi_ | l≤v,f_b_ | ns | l,v,f_b_ |
| F2 | imp30 | D_Lu_ | f_b_,l<v | D_Lu_ | l,f_b_≤v |
| F2 | imp32 | D_Vi_ | f_b_,v<l | ns | f_b_,v,l |
| F2 | imp34 | D_Vi_ | f_b_,v<l | D_Vi_ | v,f_b_≤l |
| F2 | beta | D_Lu_ | l,f_b_<v | D_Lu_ | l,f_b_<v |
| F3 | imp4 | D_Vi_ | l≤v,f_b_ | D_Vi_ | l≤v,f_b_ |
| F3 | imp6 | D_Vi_ | l<f_b_<v | D_Vi_ | l<f_b_<v |
| F3 | imp14 | D_Vi_ | l<v,f_b_ | D_Vi_ | l<v,f_b_ |
| F3 | imp16 | D_Vi_ | l<v,f_b_ | D_Vi_ | l<v<f_b_ |
| F3 | imp21 | D_Vi_ | l<v,f_b_ | ns | l,fb,v |
| F3 | imp25 | D_Vi_ | l<v,f_b_ | D_Vi_ | l<v,f_b_ |
| F4 | imp11 | D_Vi_ | f_b_,v≤l | D_Vi_ | f_b_,v<l |
| F4 | imp20 | D_Vi_ | f_b_,v<l | D_Vi_ | f_b_,v<l |
| F5 | imp8 | D_Vi_ | l<v,f_b_ | D_Vi_ | l<v,f_b_ |
| F5 | imp10 | D_Vi_ | v,f_b_<l | D_Vi_ | f_b_,v<l |
| F5 | imp13 | ns | l,f_b_,v | D_Lu_ | l,f_b_≤v |
| F5 | imp15 | D_Vi_ | l≤v,f_b_ | D_Vi_ | l≤v,f_b_ |
| F3,6 | imp2 | ns | v,l,f_b_ | D_Lu_ | v≤l,f_b_ |
| F6 | alpha | D_Vi_ | l<v,f_b_ | D_Vi_ | l<f_b_,v |
| F7 | imp5 | ns | l,f_b_,v | ns | l,f_b_,v |
| F7 | imp7 | D_Vi_ | f_b_,v≤l | ns | v,f_b_,l |
| F7 | imp9 | D_Vi_ | f_b_,v≤l | D_Vi_ | f_b_,v<l |
| F7 | imp17 | D_Vi_ | f_b_,v<l | D_Vi_ | f_b_,v<l |
| HH | imp3 | D_Vi_ | v,f_b_<l | D_Vi_ | f_b_,v<l |
| HH | imp18 | D_Vi_ | l<f_b_,v | D_Vi_ | l<v,f_b_ |
| HH | imp19 | D_Vi_ | l<f_b_,v | D_Vi_ | l<v,f_b_ |
| HH | imp23 | D_Vi_ | l≤v,f_b_ | ns | l,v,f_b_ |
| HH | imp24 | ID | l≤f_b_≤v | D_Vi_ | l<v,f_b_ |
| HH | imp27 | D_Vi_ | l<v,f_b_ | D_Vi_ | l<v,f_b_ |
| HH | imp28 | D_Lu_ | l,f_b_≤v | ns | l,f_b_,v |
