# Supplementary material for: The effects of the sex chromosomes on the inheritance of species-specific traits of the copulatory organ shape in Drosophila virilis and Drosophila lummei
Source: PLoS One. 2020 Dec 29;15(12):e0244339. doi: 10.1371/journal.pone.0244339 (PMC7771703; doi:10.1371/journal.pone.0244339)
Supplement: S3 Table — All four factors and their interactions were used as predictors; the 35 phenotypic traits, as independent variables. (DOCX) [file pone.0244339.s004.docx]

S3 Table. Significant effects and interactions as revealed by factorial MANOVA.

|  | ChrX | ChrY | Aut | ♂P | ChrY*Aut |
| --- | --- | --- | --- | --- | --- |
| F | 12.06 | 10.51 | 12.97 | 3.64 | 2.32 |
| p | 0 | 0 | 0 | 0 | 0.0009 |

All four factors and their interactions were used as predictors; the 35 phenotypic traits, as independent variables.
