# Supplementary material for: The effects of the sex chromosomes on the inheritance of species-specific traits of the copulatory organ shape in Drosophila virilis and Drosophila lummei
Source: PLoS One. 2020 Dec 29;15(12):e0244339. doi: 10.1371/journal.pone.0244339 (PMC7771703; doi:10.1371/journal.pone.0244339)
Supplement: S4 Table — Traits are grouped according to their maximal weights in the respective factors (Table 3). F, Fisher’s test; p, the significance of effects of independent variables, including X, the X chromosome; Aut, the autosomes; ♂P, the paternal genotype; and ChrY*Aut, a combined effect of the Y chromosome and autosomes. HC, a group of the traits that were not incorporated in the factor structures with weights higher than |0.5|. Significance values р < 0.05 are in bold. In each group of traits determining the respective factor structure, the lowermost row shows the estimated effects of the independent variables on the given factor. (DOCX) [file pone.0244339.s005.docx]

S4 Table. Effects of the sex chromosomes, autosomes, and parental genotypes on trait expression.

| Factor | Sign | Chr X | | Chr Y | | Aut | | ♂P | | Chr Y*Aut | |
| --- | --- | --- | --- | --- | --- | --- | --- | --- | --- | --- | --- |
|  |  | F | p | F | p | F | p | F | p | F | p |
| F1 | IMP33 | 3.38 | 0.0684 | 2.11 | 0.1489 | 9.32 | **0.0002** | 1.11 | 0.3327 | 0.13 | 0.7165 |
|  | F1 | 0.19 | 0.663 | 0.06 | 0.813 | 0.76 | 0.468 | 0.26 | 0.774 | 0.14 | 0.711 |
| F2 | IMP30 | 14.23 | **0.0002** | 1.03 | 0.312 | 2.01 | 0.1386 | 15.86 | **7.86E-07** | 0 | 0.9538 |
|  | IMP32 | 7.14 | **0.0086** | 2.66 | 0.1056 | 10.12 | **8.76E-05** | 13.68 | **4.48E-06** | 0.47 | 0.4959 |
|  | IMP34 | 2.87 | 0.0928 | 4.61 | **0.0337** | 21.32 | **1.22E-08** | 8.35 | **0.0004** | 0.87 | 0.3514 |
|  | beta | 13.08 | **0.0004** | 10.3 | **0.0017** | 0.17 | 0.8408 | 15.15 | **1.38E-06** | 0.01 | 0.9343 |
|  | F2 | 1.73 | 0.191 | 4.99 | **0.027** | .78 | 0.459 | 9.98 | **0.000** | 0.60 | 0.439 |
| F3 | IMP4 | 7.96 | **0.0056** | 4.11 | **0.0449** | 19.96 | **3.34E-08** | 2.11 | 0.12 | 0.53 | 0.4672 |
|  | IMP6 | 34.28 | **4.31E-08** | 18.93 | **2.88E-05** | 41.39 | **2.27E-14** | 6.1 | **0.003** | 5.35 | **0.0224** |
|  | IMP14 | 33.58 | **5.72E-08** | 46.78 | **3.65E-10** | 43.02 | **8.71E-15** | 0.32 | 0.7266 | 3.83 | 0.0528 |
|  | IMP16 | 87.76 | **5.87E-16** | 45.67 | **5.48E-10** | 38.5 | **1.27E-13** | 5.73 | **0.0042** | 3.89 | 0.0508 |
|  | IMP25 | 66.22 | **4.45E-13** | 36.34 | **1.91E-08** | 33.61 | **2.68E-12** | 7.93 | **0.0006** | 4.39 | **0.0383** |
|  | IMP21 | 6.55 | **0.0117** | 0.76 | 0.3863 | 12.6 | **1.09E-05** | 0.34 | 0.7145 | 3.53 | 0.0626 |
|  | F3 | 5.94 | **0.016** | 2.87 | 0.093 | 62.44 | **0.000** | 6.42 | **0.002** | 0.44 | 0.506 |
| F4 | IMP11 | 63.21 | **1.19E-12** | 3.17 | 0.0776 | 1.92 | 0.1498 | 0.68 | 0.507 | 3.34 | 0.0700 |
|  | IMP12 | 0.07 | 0.7932 | 3.04 | 0.0839 | 2.88 | 0.0597 | 0.36 | 0.7002 | 1.05 | 0.3065 |
|  | IMP20 | 73.03 | **5.04E-14** | 0 | 0.9956 | 2.56 | 0.0819 | 2.25 | 0.1102 | 3.62 | 0.0596 |
|  | F4 | 0.007 | 0.933 | 0.11 | 0.742 | 0.30 | 0.745 | 0.78 | 0.461 | 5.18 | **0.025** |
| F5 | IMP10 | 43.6 | **1.18E-09** | 22.41 | **6.12E-06** | 20.14 | **2.92E-08** | 6.62 | **0.0019** | 0.03 | 0.8557 |
|  | IMP8 | 14.24 | **0.0002** | 2.26 | 0.135 | 20.91 | **1.65E-08** | 0.14 | 0.8669 | 4.38 | **0.0386** |
|  | IMP13 | 3.34 | 0.0703 | 0.19 | 0.6662 | 5.34 | **0.006** | 6.76 | **0.0017** | 0.91 | 0.3417 |
|  | IMP15 | 0.02 | 0.8986 | 2.3 | 0.1318 | 11.37 | **3.04E-05** | 0.07 | 0.9332 | 0.04 | 0.8434 |
|  | F5 | 4.15 | **0.044** | 8.59 | **0.004** | 14.59 | **0.000** | 3.38 | **0.037** | 0.23 | 0.630 |
| F3;6 | IMP2 | 20.36 | **1.51E-05** | 0.05 | 0.8309 | 15 | **1.60E-06** | 9.01 | **0.0002** | 4.58 | **0.0343** |
| F6 | alpha | 5.28 | **0.0234** | 33.28 | **6.44E-08** | 69.6 | **< 2.2e-16** | 28.78 | **6.39E-11** | 0.03 | 0.8704 |
|  | F6 | 1.94 | 0.166 | 8.13 | **0.005** | 46.64 | **0.000** | 23.97 | **0.000** | 0.540 | 0.464 |
| F7 | IMP5 | 0.85 | 0.3586 | 3.06 | 0.0828 | 5.44 | **0.0055** | 4.65 | **0.0113** | 8.97 | **0.0033** |
|  | IMP7 | 0.02 | 0.9012 | 3.05 | 0.0831 | 7.14 | **0.0012** | 1.54 | 0.2195 | 8.14 | **0.0051** |
|  | IMP17 | 0.18 | 0.6681 | 18.11 | **4.19E-05** | 11.93 | **1.89E-05** | 6.63 | **0.0019** | 5.87 | **0.0169** |
|  | IMP9 | 5.66 | **0.0189** | 17.33 | **5.96E-05** | 0.81 | 0.4466 | 8.34 | **0.0004** | 0.21 | 0.6471 |
|  | F7 | 1.65 | 0.202 | 2.37 | 0.127 | 7.83 | **0.001** | 9.39 | **0.000** | 7.92 | **0.006** |
| HC | IMP3 | 2.34 | 0.1283 | 18.54 | **3.43E-05** | 16.02 | **6.91е-07** | 0.54 | 0.5829 | 11.34 | **0.001** |
|  | IMP18 | 4.59 | **0.0341** | 20.28 | **1.57E-05** | 3.7 | **0.0275** | 4.43 | **0.014** | 0.73 | 0.3935 |
|  | IMP19 | 0.1 | 0.7477 | 34.79 | **3.52E-08** | 4.67 | **0.0111** | 2.69 | 0.072 | 0.27 | 0.604 |
|  | IMP24 | 0.01 | 0.9266 | 18.73 | **3.15E-05** | 1.49 | 0.2297 | 7.08 | **0.0012** | 5.08 | **0.026** |
|  | IMP23 | 12.45 | **0.0006** | 0.67 | 0.4158 | 2.64 | 0.0756 | 6.48 | **0.0021** | 8.26 | **0.0048** |
|  | IMP27 | 13.35 | **0.0004** | 0.25 | 0.6199 | 3.95 | **0.0218** | 2.96 | 0.0557 | 0.94 | 0.3347 |
|  | IMP28 | 5.64 | **0.0191** | 1.3 | 0.2558 | 1.92 | 0.1508 | 7.48 | **0.0009** | 0.66 | 0.4197 |

Traits are grouped according to their maximal weights in the respective factors (Table 3). F, Fisher’s test; p, the significance of effects of independent variables, including X, the X chromosome; Aut, the autosomes; ♂P, the paternal genotype; and ChrY*Aut, a combined effect of the Y chromosome and autosomes. HC, a group of the traits that were not incorporated in the factor structures with weights higher than |0.5|. Significance values р < 0.05 are in bold. In each group of traits determining the respective factor structure, the lowermost row shows the estimated effects of the independent variables on the given factor.
