# Supplementary material for: The effects of the sex chromosomes on the inheritance of species-specific traits of the copulatory organ shape in Drosophila virilis and Drosophila lummei
Source: PLoS One. 2020 Dec 29;15(12):e0244339. doi: 10.1371/journal.pone.0244339 (PMC7771703; doi:10.1371/journal.pone.0244339)
Supplement: S5 Table — (+), a significant effect of a hereditary factor that is in linear relationship with the given genotype classes; (+*), a significant effect of a hereditary factor that is in nonlinear relationship with the given genotype classes; DVi (Lu), dominance of the D. virilis (D. lummei) phenotype in genotypes with the intermediate value of the indicator variable; PY(Aut), an epigenetic effect of the male parent identity on the Y chromosome (autosomes); the subscripts Aut, X-chr, and Y-chr indicate that the autosome set, X chromosome, or Y chromosome determines phenotype dominance; F#, latent trait number as in Table 3; F#*, permutation test values for the factors (latent traits) used as variables; CH, highly characteristic trait; FDR, false discovery rate for the primary traits. (DOCX) [file pone.0244339.s006.docx]

S5 Table. Permutational ANOVA to separate the genotypes into groups homogeneous in trait values.

| *F##* | *Signs* |  | *X_Y (epist)* | *AUT (add)* | *X_P + AUT (dom)_P* | *Y→AUT (dom. epist) + Y* | *AUT (add)*_*P + Y_P* | *Y→AUT (rec.epist) + X→AUT (rec.epist)* | *X→AUT (dom. epist) + X+AUT (dom)* |
| --- | --- | --- | --- | --- | --- | --- | --- | --- | --- |
| F1 | IMP29 | Prob. | 0.355 | 0.160 | 0.508 | 0.966 | 0.217 | 0.339 | 0.115 |
|  |  | Dom. | n.s. | n.s. | n.s. | n.s. | n.s. | n.s. | n.s. |
|  | IMP31 | Prob. | 0.351 | 0.804 | 0.012 | 0.150 | 0.091 | 0.844 | 0.051 |
|  |  | Dom. | n.s. | n.s. | +* sD^Vi^_Aut_ | n.s. | n.s. | n.s. | n.s. |
|  | IMP33 | Prob. | 0.185 | 0.039 | 0.000 | 0.050 | 0.002 | 0.582 | 0.002 |
|  |  | Dom. | n.s. | n.s. | + D^Vi^_Aut_ | n.s. | +* D^Vi^_Aut, (X?)_ | n.s. | +* sD^Vi^_Aut_ |
| F1* |  | Prob.* | 0.493 | 0.476 | 0.473 | 0.136 | 0.302 | 0.638 | 0.304 |
|  |  | Dom.* | n.s. | n.s. | n.s. | n.s. | n.s. | n.s. | n.s. |
| F2 | IMP30 | Prob. | 0.007 | 0.160 | 0.000 | 0.480 | 0.000 | 0.011 | 0.004 |
|  |  | Dom. | + | n.s. | + D^Lu^_X-chr_ | n.s. | + P_Y_ | + ID | +* sD^Lu^_X-chr*_ |
|  | IMP32 | Prob. | 0.157 | 0.078 | 0.000 | 0.023 | 0.000 | 0.062 | 0.000 |
|  |  | Dom. | n.s. | n.s. | + sD^Lu^_X-chr_ | n.s. | *_(X-chr)_ | n.s. | +* |
|  | IMP34 | Prob. | 0.103 | 0.000 | 0.000 | 0.025 | 0.000 | 0.025 | 0.000 |
|  |  | Dom. | n.s. | D^Vi^ | +* | n.s. | + D^Vi^_Aut_ | +* sD^Lu^_Y-chr_ | + sD^Vi^_Aut_ |
|  | beta* | Prob. | 0.001 | 0.007 | 0.000 | 0.331 | 0.000 | 0.000 | 0.003 |
|  |  | Dom. | + | ID | + D^Lu^_X-chr_ | n.s. | + D^Lu^_Aut_ | + D^Lu^_Y-chr_ | + ID |
| F2* |  | Prob.* | 0.113 | 0.207 | **0.000** | **0.038** | **0.000** | 0.057 | **0.000** |
|  |  | Dom.* | n.s. | n.s. | + sD^Lu^_X-chr_ | + | *_(X-chr)_ | n.s. | +*(sD^Vi^_X-chr*_) |
| F3 | IMP4 | Prob. | 0.008 | 0.000 | 0.077 | 0.675 | 0.022 | 0.000 | 0.051 |
|  |  | Dom. | + | D^Lu^ | n.s. | n.s. | + D^Lu^_Aut_ | + D^Vi^_X-chr_ | n.s. |
|  | IMP6 | Prob. | 0.000 | 0.000 | 0.015 | 0.446 | 0.000 | 0.000 | 0.000 |
|  |  | Dom. | + | ID | +* sD^Vi^_Aut_ | n.s. | + P_Y_ | + D^Vi^_X-chr_ | + D^Lu^_X-chr*_ |
|  | IMP14 | Prob. | 0.000 | 0.000 | 0.074 | 0.020 | 0.000 | 0.000 | 0.000 |
|  |  | Dom. | + | ID | n.s. | n.s. | + P_Aut_ | + D^Vi^_X-chr_ | + D^Vi^_Aut_ |
|  | IMP16 | Prob. | 0.000 | 0.000 | 0.006 | 0.320 | 0.000 | 0.000 | 0.000 |
|  |  | Dom. | + | ID | +* | n.s. | + P_Aut_ | + D^Vi^_X-chr_ | + ID |
|  | IMP21 | Prob. | 0.246 | 0.000 | 0.279 | 0.722 | 0.058 | 0.000 | 0.049 |
|  |  | Dom. | n.s. | D^Lu^ | n.s. | n.s. | n.s. | +* sD^Vi^_X-chr_ | n.s. |
|  | IMP25 | Prob. | 0.000 | 0.000 | 0.000 | 0.311 | 0.000 | 0.000 | 0.000 |
|  |  | Dom. | + | ID | + D^Lu^_X-chr_ | n.s. | + P_Aut_ | + D^Vi^_X-chr_ | + ID |
| F3* |  | Prob.* | **0.001** | **0.000** | **0.000** | 0.285 | **0.000** | **0.000** | **0.000** |
|  |  | Dom.* | + | D^Lu^ | +* | n.s. | + P_Y_, D^Lu^_Aut_ | +* sD^Vi^_X-chr_ | +* sD^Lu^_X-chr*_ |
| F4 | IMP11 | Prob. | 0.100 | 0.021 | 0.000 | 0.000 | 0.000 | 0.019 | 0.000 |
|  |  | Dom. | n.s. | D^Lu^ | +* | + | + P_Y_ | + ID | + D^Lu^_X-chr*_ |
|  | IMP20 | Prob. | 0.041 | 0.000 | 0.000 | 0.001 | 0.000 | 0.001 | 0.000 |
|  |  | Dom. | + | ID | +* | + | + P_Y_ | + D^Vi^_X-chr_ | + D^Lu^_X-chr*_ |
| F4* |  | Prob.* | 0.697 | 0.489 | **0.000** | **0.000** | **0.000** | 0.738 | **0.000** |
|  |  | Dom.* | n.s. | n.s. | +* | + | + P_Y_ | n.s. | +* sD^Lu^_X-chr*_ |
| F5 | IMP8 | Prob. | 0.431 | 0.000 | 0.347 | 0.739 | 0.000 | 0.696 | 0.000 |
|  |  | Dom. | n.s. | D^Vi^ | n.s. | n.s. | + D^Vi^_Aut_ | n.s. | + D^Vi^_Aut_ |
|  | IMP10 | Prob. | 0.000 | 0.000 | 0.000 | 1.000 | 0.000 | 0.000 | 0.000 |
|  |  | Dom. | + | D^Lu^ | +* | n.s. | +* D^Lu^_Aut_ | + D^Vi^_X-chr_ | + D^Lu^_X-chr_ |
|  | IMP13 | Prob. | 0.097 | 0.278 | 0.000 | 0.260 | 0.023 | 0.964 | 0.011 |
|  |  | Dom. | n.s. | n.s. | + D^Vi^_Aut_ | n.s. | +* D^Lu,Vi^_Aut_ | n.s. | +* |
|  | IMP15 | Prob. | 0.002 | 0.031 | 0.368 | 0.177 | 0.013 | 0.020 | 0.031 |
|  |  | Dom. | + | sD^Vi^ | n.s. | n.s. | +* D^Vi^_Aut_ | +* sD^Lu^_Y-chr_ | +* sD^Vi^_Aut_ |
| F5* |  | Prob.* | **0.000** | **0.000** | 0.043 | 0.025 | **0.001** | **0.000** | 0.051 |
|  |  | Dom.* | + | +* | n.s. | + | +* D^Lu^_Aut_ | + ID | n.s. |
| F3, F6 | IMP2 | Prob. | 0.001 | 0.000 | 0.000 | 0.121 | 0.000 | 0.001 | 0.000 |
|  |  | Dom. | + | sD^Lu^ | + ID | n.s. | *_X-chr_ | + D^Vi^_X-chr_ | +* |
| F6 | alpha | Prob. | 0.717 | 0.000 | 0.000 | 0.016 | 0.000 | 0.189 | 0.000 |
|  |  | Dom. | n.s. | D^Vi^ | + D^Vi^_Aut_ | + | + D^Vi^_Aut_ | n.s. | +* sD^Vi^_Aut_ |
| F6* |  | Prob.* | 0.478 | **0.000** | **0.000** | **0.000** | **0.000** | 0.857 | **0.000** |
|  |  | Dom.* | n.s. | D^Vi^ | + D^Vi^_Aut_ | + | +D^Vi^_Aut_ (*_X-chr_) | n.s. | + ID |
| F7 | IMP5 | Prob. | 0.026 | 0.011 | 0.092 | 0.078 | 0.043 | 0.044 | 0.382 |
|  |  | Dom. | + | sD^Lu^ | n.s. | n.s. | n.s. | n.s. | n.s. |
|  | IMP7 | Prob. | 0.804 | 0.002 | 0.122 | 0.150 | 0.017 | 0.036 | 0.003 |
|  |  | Dom. | n.s. | sD^Vi^ | n.s. | n.s. | + D^Vi^_Aut_ | n.s. (D^Vi^_X-chr_) | + D^Vi^_Aut_ |
|  | IMP9 | Prob. | 0.001 | 0.001 | 0.220 | 0.023 | 0.001 | 0.005 | 0.004 |
|  |  | Dom. | + | ID | n.s. | n.s. | + D^Vi^_Aut_ | + ID | + ID |
|  | IMP17 | Prob. | 0.440 | 0.000 | 0.010 | 0.001 | 0.000 | 0.547 | 0.000 |
|  |  | Dom. | n.s. | D^Vi^ | +* sD^Lu^_X-chr_ | + | + D^Vi^_Aut_ | n.s. | + D^Vi^_Aut_ |
| F7* |  | Prob.* | 0.542 | **0.000** | **0.045** | **0.031** | **0.002** | **0.028** | **0.001** |
|  |  | Dom.* | n.s. | sD^Vi^ | n.s.(sD^Lu^_X-chr_) | + | +* D^Vi^_Aut_ | +* | + D^Vi^_Aut_ |
| CH | IMP3 | Prob. | 0.000 | 0.000 | 0.811 | 0.009 | 0.000 | 0.000 | 0.123 |
|  |  | Dom. | + | D^Lu^ | n.s. | + | + D^Lu^_Aut_ | + D^Vi^_X-chr_ | n.s. |
|  | IMP18 | Prob. | 0.000 | 0.000 | 0.222 | 0.008 | 0.009 | 0.000 | 0.085 |
|  |  | Dom. | + | D^Lu^ | n.s. | + | + P_Aut_ | + D^Vi^_X-chr_ | n.s. |
|  | IMP19 | Prob. | 0.000 | 0.000 | 0.302 | 0.000 | 0.002 | 0.000 | 0.009 |
|  |  | Dom. | + | ID | n.s. | + | + D^Vi^_Aut_ | + ID | + D^Vi^_Aut_ |
|  | IMP23 | Prob. | 0.385 | 0.004 | 0.033 | 0.412 | 0.002 | 0.000 | 0.002 |
|  |  | Dom. | n.s. | D^Vi^ | n.s. | n.s. | +* D^Vi^_Aut_ | +* | + ID |
|  | IMP24 | Prob. | 0.010 | 0.004 | 0.000 | 0.000 | 0.004 | 0.003 | 0.016 |
|  |  | Dom. | + | D^Vi^ | + D^Vi^_Aut_ | + | + D^Vi^_Aut_ | + ID | +* sD^Vi^_Aut_ |
|  | IMP26 | Prob. | 0.055 | 0.000 | 0.239 | 1.000 | 0.145 | 0.000 | 0.120 |
|  |  | Dom. | n.s. | D^Lu^ | n.s. | n.s. | n.s. | + D^Vi^_X-chr_ | n.s. |
|  | IMP27 | Prob. | 0.306 | 0.000 | 0.054 | 0.253 | 0.004 | 0.000 | 0.002 |
|  |  | Dom. | n.s. | D^Lu^ | n.s. | n.s. | +*_X-chr_ | +* sD^Vi^_X-chr_ | + D^Lu^_X-chr*_ |
|  | IMP28 | Prob. | 0.020 | 0.538 | 0.000 | 1.000 | 0.002 | 0.042 | 0.067 |
|  |  | Dom. | + | n.s. | + D^Lu^_X-chr_ | n.s. | + D^Lu^_Aut_ | n.s. | n.s. |
| FDR | 0.05 | | 0.023 | 0.037 | 0.026 | 0.010 | 0.040 | 0.031 | 0.034 |
|  | 0.01 | | 0.003 | 0.006 | 0.004 | 0.001 | 0.007 | 0.005 | 0.006 |

(+), a significant effect of a hereditary factor that is in linear relationship with the given genotype classes; (+*), a significant effect of a hereditary factor that is in nonlinear relationship with the given genotype classes; D^Vi (Lu)^, dominance of the *D. virilis* (*D. lummei*) phenotype in genotypes with the intermediate value of the indicator variable; P_Y(Aut)_, an epigenetic effect of the male parent identity on the Y chromosome (autosomes); the subscripts Aut, X-chr, and Y-chr indicate that the autosome set, X chromosome, or Y chromosome determines phenotype dominance; F#, latent trait number as in Table 3; F#*, permutation test values for the factors (latent traits) used as variables; CH, highly characteristic trait; FDR, false discovery rate for the primary traits.
