# Supplementary material for: The effects of the sex chromosomes on the inheritance of species-specific traits of the copulatory organ shape in Drosophila virilis and Drosophila lummei
Source: PLoS One. 2020 Dec 29;15(12):e0244339. doi: 10.1371/journal.pone.0244339 (PMC7771703; doi:10.1371/journal.pone.0244339)
Supplement: S6 Table — (DOCX) [file pone.0244339.s007.docx]

S6 Table. Homogeneous groups in latent trait (F_n_) variance identified by the *post hoc* tests.

|  | *X→Y (epist)* | | *AUT (add)* | | | *P→X+P→AUT (dom)* | | | *Y+Y→AUT*  *(dom.epist)* | | *P→AUT (add)+P→Y* | | | | *Y→AUT (rec.epist)+ X→AUT (rec.epist)* | | | *X→AUT (dom.epist) +X+AUT (dom)* | | |
| --- | --- | --- | --- | --- | --- | --- | --- | --- | --- | --- | --- | --- | --- | --- | --- | --- | --- | --- | --- | --- |
| А_i_ | 0 | 1 | -1 | 0 | 1 | 0 | 1 | 2 | 0 | 2 | -1 | 0 | 1 | 2 | 0 | 1 | 2 | 0 | 1 | 3 |
| F1 | a | a | a | a | a | a | a | a | a | a | a | a | a | a | a | a | a | a | a | a |
|  | -0.03 | 0.14 | 0.29 | -0.08 | 0.14 | -0.01 | 0.2 | -0.15 | -0.13 | 0.17 | 0.29 | -0.17 | 0.2 | 0.24 | -0.04 | -0.03 | 0.23 | 0.29 | 0.2 | -0.1 |
| F2 | a | a | a | a | a | a | b | c | a | b | a | a | b | a | ab | a | b | a | b | a |
|  | -0.07 | 0.32 | 0.57 | -0.06 | -0 | -0.1 | -0.64 | 0.67 | 0.17 | -0.23 | 0.57 | 0.13 | -0.64 | 0.36 | 0 | -0.65 | 0.36 | 0.57 | -0.64 | 0.16 |
| F3 | a | b | a | a | b | a | b | a | a | a | ab | ac | b | c | a | b | c | ab | a | b |
|  | -0.14 | 0.65 | -0.38 | -0.4 | 1.39 | 0.35 | -0.68 | 0.02 | 0.08 | -0.11 | -0.38 | 0.22 | -0.68 | 0.69 | -0.39 | 2.08 | 1.01 | -0.38 | -0.68 | 0.29 |
| F4 | a | a | a | a | a | a | b | a | a | b | ab | a | b | a | a | a | a | ab | a | b |
|  | 0.02 | -0.07 | 0.36 | -0.02 | -0.07 | -0.22 | 0.77 | -0.29 | -0.3 | 0.41 | 0.36 | -0.37 | 0.77 | -0.02 | 0.02 | 0.1 | -0.16 | 0.36 | 0.77 | -0.32 |
| F5 | a | b | a | b | a | a | a | a | a | b | ab | a | a | b | a | ab | b | a | a | a |
|  | 0.2 | -0.92 | -0.64 | 0.26 | -0.59 | -0.25 | 0.26 | 0.17 | 0.18 | -0.24 | -0.64 | 0.13 | 0.26 | -0.88 | 0.17 | -0.07 | -0.88 | -0.64 | 0.26 | -0.02 |
| F6 | a | a | a | b | b | a | b | b | a | b | a | b | c | bc | a | a | a | a | b | c |
|  | 0.03 | -0.14 | -2.27 | 0.22 | 0.1 | -0.72 | 0.73 | 0.51 | -0.27 | 0.36 | -2.27 | -0.06 | 0.73 | 0.37 | -0.03 | 0.13 | 0.08 | -2.27 | 0.73 | 0 |
| F7 | a | a | a | b | ab | ab | a | b | a | b | a | b | b | ab | a | b | ab | a | b | b |
|  | 0.02 | -0.11 | 1.03 | -0.19 | 0.22 | -0.02 | -0.3 | 0.28 | 0.16 | -0.22 | 1.03 | -0.03 | -0.3 | 0.09 | -0.06 | 0.78 | -0.09 | 1.03 | -0.3 | -0.01 |

F_n_, latent trait number as in Table 3; А_i_ is the indicator variable for the groups of genotypes in accordance with the effects of hereditary factors (Table 2); a, b, and c designate homogeneous groups in latent trait variance; numbers in cells present mean values of the latent traits.
