# Supplementary material for: The effects of the sex chromosomes on the inheritance of species-specific traits of the copulatory organ shape in Drosophila virilis and Drosophila lummei
Source: PLoS One. 2020 Dec 29;15(12):e0244339. doi: 10.1371/journal.pone.0244339 (PMC7771703; doi:10.1371/journal.pone.0244339)
Supplement: S1 Text — (DOCX) [file pone.0244339.s008.docx]

**S1 Text. Additional material for the last subsection “The role of the components of variability and their combinations in the inheritance of traits of the shape of the male copulative organ” of the Result section**

Factor X→Y(epist) has two categories for the A_1_ indicator variable, the effect being present or absent. A significant difference in these categories between samples confirms the significant effect of the interaction between the sex chromosomes. The effect of the conspecific *D. virilis* sex chromosomes is examined versus the effects of all other combinations in this case.

Factor Y+Y→AUT(dom.epist) has two similar categories for the A_5_ indicator variable. The independent effect of the Y chromosome alone on the traits is close to zero. Therefore, a significant difference in the categories between samples confirms the significant effect of the interaction between the Y chromosome and autosomes. The effect of the *D. virilis* Y chromosome and autosomes is examined versus the effects of all other combinations in this case.

With all subsequent factors, a significant difference between the extreme variants confirms that the respective factor significantly affects the trait in question. A significant difference between intermediate and other genotype groups in the absence of differences between the extreme groups indicates that the effect in question depends nonlinearly on the genotype.

Factor AUT(add) has three categories of the A_2_ indicator variable: two opposite effects of the *D. virilis* and *D. lummei* homozygous autosomes and an intermediate effect of the heterozygous autosomes. An intermediate effect suggests a decrease in additive interactions of divergent genes, and the lack of significant difference from one of the homozygous genotypes indicates that dominant alleles mostly contribute to the trait expression.

Factor Y→AUT(rec.epist)+X→AUT(rec.epist) has three categories of the A_4_ indicator variable: an effect of interactions between the homozygous *D. virilis* autosomes and the *D. virilis* sex chromosomes, a partial effect of interactions between the homozygous *D. virilis* autosomes and the X chromosome, and all variants with the heterozygous autosomes or homozygous *D. lummei* autosomes. A significant difference between the extreme variants indicates that the sex chromosomes interact with recessive autosomal genes, leading to species-specific distinctions. Lack of a significant difference from one of the extreme variants suggests a predominant contribution of epistatic interactions with one of the sex chromosomes, depending on the genotype combination (dominance of the *D. virilis* phenotype suggests a leading role for the X chromosome; dominance of the *D. lummei* phenotype, for the *D. virilis* Y chromosome, which is absent in 1.9.30 males, of the *D. lummei* Y chromosome present in the given genotype).

Factor P→X+P→AUT(dom) has three categories of the A_6_ indicator variable: the minimal and maximal values correspond, respectively, to the absence or presence of interactions of the *D. virilis* X chromosome and autosomes with the homozygous paternal genotype, and an intermediate value corresponds to interactions of dominant genes of the *D. virilis* autosomes with the *D. virilis* homozygous paternal genotype. A grouping of genotypes having intermediate values with those having one of the extreme values maximizes the role of the autosomes or the X chromosome, depending on the group composition (dominance of the *D. virilis* phenotype suggests a maximal role for the autosomes; dominance of the *D. lummei* phenotype, for the *D. virilis* X chromosome, which is absent in (*P_Vi_*)*F_1_* *X*_Lu_*Y*_Vi_A_Vi/Lu_ males).

Factor X→AUT(dom.epist)+X+AUT(dom) has three categories of the A_7_ indicator variable. The minimal and maximal values correspond to expression of the *D. virilis* phenotype due to the effects of dominant autosomal genes and the *D. virilis* X chromosome and their epistatic interactions. The intermediate value corresponds to a sole effect of dominant autosomal genes. Dominance of the *D. virilis* phenotype (a grouping of genotype *X*_Lu_*Y*_Vi_ Aut_Vi/Lu_ with genotypes X*_Vi_*Y*Aut*_Vi_*_/*_) suggests a predominant effect for *D. virilis* dominant autosomal genes; dominance of the *D. lummei* phenotype, for the *D. lummei* X chromosome, *D. lummei* autosomes, or their combination.

Factor P→AUT(add)+P→Y has four categories of the A_3_ indicator variable. The extreme values define the effects that the paternal genotype exerts on the expression of the *D. virilis* phenotype under the influence of recessive autosomal alleles and the Y chromosome. The two alternative extreme values accordingly belong to the genotypes of the *D. virilis* and *D. lummei* parental strains. The intermediate values of indicator variables are defined by the epigenetic effect that the *D. virilis* male parent identity exerts exclusively on the *D. virilis* Y chromosome (genotype (*P_Vi_*)*F_1_* *X*_Lu_*Y*_Vi_A_Vi/Lu_, the value is 1) and by lack of this effect on the genotypes that are heterozygous for the autosomes and have the Y chromosome originating from a heterozygous male or a *D. lummei* male (genotypes (*P_Lu_*)*F_1_ X*_Vi_*Y*_Lu_A_Vi/Lu_, (*P_Vi/Lu_*)*F_b_* *X*_Vi_*Y*_Vi_A_Vi/Lu_, (*P_Vi/Lu_*)*F_b_* *X*_Vi_*Y*_Vi_A_Vi_, (*P_Vi/Lu_*)*F_b_* *X*_Vi_*Y*_Lu_A_Vi/Lu_, and (*P_Vi/Lu_*)*F_b_* *X*_Vi_*Y*_Lu_A_Vi_; the value is 0). Lack of a significant epigenetic effect on the autosomes will lead to the clustering of the *D. lummei* genotype with genotype (*P_Vi_*)*F_1_* *X*_Lu_*Y*_Vi_A_Vi/Lu_ and the *D. virilis* genotype with genotypes (*P_Lu_*)*F_1_* *X*_Vi_*Y*_Lu_A_Vi/Lu_, (*P_Vi/Lu_*)*F_b_* *X*_Vi_*Y*_Vi_A_Vi/Lu_, (*P_Vi/Lu_*)*F_b_* *X*_Vi_*Y*_Vi_A_Vi_, (*P_Vi/Lu_*)*F_b_* *X*_Vi_*Y*_Lu_A_Vi/Lu_, and (*P_Vi/Lu_*)*F_b_* *X*_Vi_*Y*_Lu_A_Vi_. If a significant epigenetic or genetic effects of the Y chromosome is lacking, the genotype (*P_Vi_*)*F_1_* *X*_Lu_*Y*_Vi_A_Vi/Lu_ will cluster with genotypes (*P_Lu_*)*F_1_* *X*_Vi_*Y*_Lu_A_Vi/Lu_, (*P_Vi/Lu_*)*F_b_* *X*_Vi_*Y*_Vi_A_Vi/Lu_, (*P_Vi/Lu_*)*F_b_* *X*_Vi_*Y*_Vi_A_Vi_, (*P_Vi/Lu_*)*F_b_* *X*_Vi_*Y*_Lu_A_Vi/Lu_, and (*P_Vi/Lu_*)*F_b_* *X*_Vi_*Y*_Lu_A_Vi_. If the autosomes exert a dominant effect characteristic of *D. virilis* or *D. lummei* in this case, strains with the intermediate values of indicator variables will cluster together with the respective parental genotype. The incomplete design of crosses makes the results difficult to interpret. For example, male genotype (*P_Vi_*)*F_1_* *X*_Lu_*Y*_Vi_A_Vi/Lu_ was the only genotype that had the intermediate value 1 for the indicator variables A3 (factor P→AUT(add)+P→Y), A6 (factor P→X+P→AUT(dom)), and A7 (factor X→AUT(dom.epist) +X+AUT(dom)). It is clear that the phenotypic features of this genotype are determined by combined effects of the three factors, and its clustering with other genotypes may therefore be distorted in the case of factor P→AUT(add)+P→Y.
